# The effect of intraoperative goal-directed crystalloid versus colloid administration on perioperative inflammatory markers - a substudy of a randomized controlled trial

**DOI:** 10.1186/s12871-020-01126-3

**Published:** 2020-08-21

**Authors:** Mina Obradovic, Andrea Kurz, Barbara Kabon, Georg Roth, Oliver Kimberger, Oliver Zotti, Ahamed Bayoumi, Christian Reiterer, Anton Stift, Edith Fleischmann

**Affiliations:** 1 Department of Anaesthesia, General Intensive Care Medicine and Pain Medicine, Medical University of Vienna, Spitalgasse 23, 1090 Vienna, Austria; 2 Department of Outcomes Research and General Anesthesiology, Anesthesiology Institute, 9500 Euclid Avenue, Cleveland Clinic, Cleveland, OH USA; 3 Department of Anesthesiology and General Intensive Care, Franziskus Hospital, Nikolsdorfergasse 32, 1050 Vienna, Austria; 4 Department of Gynecology, Klinik Ottakring, Montleartstrasse 37, 1160 Vienna, Austria; 5 Department of Surgery, Medical University of Vienna, Spitalgasse 23, 1090 Vienna, Austria

## Abstract

**Background:**

Excessive perioperative fluid administration may result in iatrogenic endothelial dysfunction and tissue edema, transducing inflammatory markers into the bloodstream. Colloids remain longer in the circulation, requiring less volume to reach similar hemodynamic endpoints compared to crystalloids. Thus, we tested the hypothesis that a goal-directed colloid regimen attenuates the inflammatory response compared to a goal-directed crystalloid regime.

**Methods:**

Patients undergoing moderate- to high-risk open abdominal surgery were randomly assigned to goal-directed lactated Ringer’s solution (*n* = 58) or a hydroxyethyl starch 6% 130/0.4 (*n* = 62) fluid regimen. Our primary outcome was perioperative levels of pro- and anti-inflammatory cytokines. Secondary outcome was perioperative levels of white blood cell count (WBC), C-reactive protein (CRP), procalcitonin (PCT) and lipopolysaccharide-binding protein (LBP). Measurements were performed preoperatively, immediate postoperatively, on postoperative day one, two and four.

**Results:**

The areas under the curve of Interleukin (IL) 6 (*p* = 0.60), IL 8 (*p* = 0.46), IL 10 (*p* = 0.68) and tumor necrosis factor α (*p* = 0.47) levels did not differ significantly between the groups. WBC, CRP and PCT values were also comparable. LBP, although significantly higher in the crystalloid group, remained in the normal range. Patients assigned to crystalloids received a median (IQR) amount of 3905 mL (2880–5288) of crystalloid. Patients assigned to colloids received 1557 mL (1207–2116) of crystalloid and 1250 mL (750–1938) of colloid.

**Conclusion:**

Cytokine and inflammatory marker levels did not differ between goal-directed crystalloid and colloid administration after moderate to high-risk abdominal surgery.

**Trial registration:**

ClinicalTrials.gov (NCT00517127). Registered 16th August 2007.

## Introduction

Volume replacement is crucial in the perioperative period and has great impact on postoperative outcome [1]. Fluid restriction may cause hypotension and hypoperfusion, leading to organ dysfunction [2]. On the other hand, excessive fluid administration leads to destruction of the endothelial surface layer and consequently to tissue edema with harmful side effects [1, 3, 4].

Goal-directed fluid therapy (GDT), based on optimization of flow-related hemodynamic parameters improves clinical outcome in low to high-risk surgical patients compared to fixed fluid protocols [5, 6]. Specifically, GDT enhances cardiac performance and gut microcirculation while avoiding iatrogenic hyperhydration [1, 7, 8]. In addition to hypervolemia, the inflammatory response due to surgical trauma aggravates degradation of the endothelial barrier, the so-called glycocalyx [9]. Inflammation leads to cytokine release and may thus worsen outcome. For example, high postoperative interleukin (IL) 6 levels are independently associated with postoperative complications [10].

So far in most previously performed GDT studies hemodynamic algorithms were based on colloid bolus administration to improve hemodynamic variables [11]. Colloids better maintain the intravascular oncotic pressure and provide a higher volume effect when used in case of hypovolemia [12]. Goal-directed colloid administration reduces intraoperative fluid requirement and improves cardiac performance compared to crystalloids [13, 14]. Whether this translates into better outcome, specifically in a decreased postoperative inflammatory response, is still a matter of research. The comparison between colloid versus crystalloid based fluid regimens was still lacking. Therefore, we tested the primary hypothesis that perioperative levels of pro- and anti-inflammatory cytokines (IL 6, IL 8, IL 10) and tumor necrosis factor alpha (TNF α) are reduced by goal-directed colloid versus crystalloid administration during the first four postoperative days in patients undergoing moderate to high-risk open abdominal surgery. In addition, we measured white blood cell (WBC) count, C-reactive protein (CRP), procalcitonin (PCT) and lipopolysaccharide-binding protein (LBP) levels.

## Materials and methods

This prospective randomized controlled trial was conducted at the Department of Anesthesia, Intensive Care Medicine and Pain Medicine, Medical University of Vienna, Vienna, Austria. The Institutional Review Board of the Medical University of Vienna approved it as part of a large multicenter outcome trial, evaluating the effect of goal-directed crystalloid and colloid on postoperative combined morbidity and complications [15]. The Ethical Committee of Medical University of Vienna, Vienna, Austria provided ethical approval for this trial. The trial was conducted in accordance with the Declaration of Helsinki and Good Clinical Practice and registered at ClinicalTrials.gov. (NCT00517127 and EudraCT: 2005–004602-86). A written informed consent was obtained from all patients. The authors have followed the applicable CONSORT guidelines.

For this single center sub-study 120 consecutive eligible patients were included. Patients aged 18 to 80 years, undergoing elective moderate to high-risk open abdominal surgery with American Society of Anesthesiologists (ASA) physical status I-III were included. We excluded patients with severe obesity (body mass index (BMI) > 35 kg.m^− 2^), cardiac insufficiency (ejection fraction (EF) < 35%), coronary artery disease with angina, severe chronic obstructive pulmonary disease, autoimmune diseases, coagulopathies, renal insufficiency (creatinine clearance < 30 ml.min^− 1^ or renal replacement therapy), symptoms of infection or sepsis and preoperative CRP higher than 1 mg.dl^− 1^.

### Protocol

Preoperatively all patients received antimicrobial prophylaxis using a single dose of a 2nd generation cephalosporine according to our clinical standards. Anesthetic management was standardized. Standard monitoring included electrocardiography (ECG), invasive blood pressure surveillance, pulse oximetry and esophageal core temperature monitoring. A central venous catheter was inserted when deemed clinically necessary. We used balanced anesthesia with sevoflurane. None of our patients received locoregional anesthesia. According to patients’ requirements additional fentanyl and non-depolarizing neuromuscular blocking were administered. Ventilatory rate was adjusted to maintain end-tidal carbon dioxide partial pressure (etCO_2_) of 35–40 mmHg. Normothermia was maintained with forced air warming.

Patients were randomized 1:1 to crystalloid (lactated Ringer’s solution) or colloid (hydroxyethyl starch 6% 130/0.4, Voluven, Fresenius Kabi, Germany) group. Randomization was based on computer-generated codes. To conceal allocation, sealed opaque envelopes were opened only shortly before induction of anesthesia.

All patients were given 5–7 ml.kg^− 1^ of lactated Ringer’s solution during induction of anesthesia followed by 3–5 ml.kg^− 1^ per hour for maintenance, normalized to ideal body weight (IBW), throughout surgery. We calculated IBW according to the Robinson formula [16]. Thereafter, the randomized fluid, crystalloid or colloid, was esophageal Doppler-guided (Cardiac Q, Deltex Medical Group PLC, Chichester, UK) according to a standard algorithm [11]. This method is based on corrected aortic flow time (FTc) as well as stroke volume (SV) and allows distinguishing whether a patient is a fluid responder or not. If mean arterial pressure (MAP) was below 65 mmHg and no signs of hypovolemia were detected, vasopressors were administrated.

Patients were transferred to post-anesthetic care unit (PACU) or intensive care unit (ICU) at the discretion of the attending anesthesiologist. Fluid management was standardized for the first 2 postoperative hours, in which patients received 2 ml.kg^− 1^ IBW crystalloid per hour.

### Measurements

Demographic and morphometric data were recorded as well as ASA score, medical history, type of surgery and preoperative laboratory values. Duration of anesthesia and surgery were recorded. We also recorded intraoperative fluid requirements, estimated blood loss, transfusion requirements and urinary output. For evaluation of anesthetic management, the total amount of fentanyl, end-tidal sevoflurane concentration, core temperature and postoperative ICU admission were noted. Hemodynamic parameters such as MAP, heart rate (HR), FTc, SV and cardiac output (CO) were recorded at 10-min intervals. The application of phenylephrine use was noted.

The primary outcomes were the areas under the curve (AUCs) of postoperative levels of pro- and anti-inflammatory cytokines IL 6, IL 8, IL 10 and TNF α and their differences between the crystalloid and the colloid group. Secondary outcomes were AUCs of WBC, CRP, PCT and LPB and their differences between the groups. All blood samples for parameter-analyses were obtained before surgery as baseline values (T0), immediately postoperatively (T1) as well as on postoperative days one, two and four (T2, T3 and T4), respectively. For analysis of IL 6, IL 8, IL 10 and TNF α blood samples were centrifuged within 1 h at 1500 G for 15 min and plasma was immediately stored at − 80 °C for later enzyme-linked immunosorbent assay (ELISA) analyses. The serum concentrations of IL 6, IL 8, IL 10 and TNF *α* were determined according to the manufacturer’s instructions (Human sIL-6 Instant ELISA, Human IL-8/NAP-1 Instant ELISA and Human sIL-10 Instant ELISA, eBioscience, Vienna, Austria, www.ebioscience.com, Human TNF α DuoSet, R&D Systems, Minneapolis, Minnesota, www.rndsystems.com). For that purpose, optical density was measured with a Victor 3 microplate reader at a wavelength of 450 nm. Multiple testing of samples on different plates revealed an intra-assay variability of 2% for IL 6, 2% for IL 8, 3% for IL 10, 1% for TNF α and an inter-assay variability of 1% for IL 6, 3% for IL 8, 2% for IL 10 and 2% for TNF α.

For investigation of WBC, CRP, PCT and LPB separate blood samples were obtained. Their analysis took place immediately after blood sampling as routine laboratory analyses.

### Sample size calculation and statistical analysis

Sample size calculations for our trial were based on the study of Steppan and colleagues [17]. They observed a mean (standard deviation, SD) of 74 (50) pg.ml^− 1^ in IL 6 24 h after surgery in 28 abdominal surgery patients. Assuming a similar coefficient of variation (SD/mean = 0.67) for each of the four cytokines primarily planned for evaluation in our study, we calculated a total of 120 patients in order to obtain a 80% power to detect a 30% reduction in any of the cytokines at an overall 0.05 significance level with 80% power.

Groups were primarily compared for balance in patients’ demographic data, intra-operative characteristics and postoperative variables. Absolute standardized differences (ASD) were calculated for patients’ baseline covariates. Subsequent measurements of intra-operative parameters were first averaged within each patient and then averaged among the patients in each treatment group for descriptive analysis. Normal distribution was assessed with q-q plots and Kolmogorow-Smirnow tests. Normally distributed variables were with unpaired, two-tailed t-tests, otherwise the in case of normally distributed values. Wilcoxon rank-sum test was used for not normally distributed continuous data. Paired comparisons between baseline data and postoperative data were performed with paired sample t-test or Wilcoxon signed-rank test, as applicable. Nominal data were analyzed with chi-square or Fisher’s exact test for low expected cell counts. Data were presented as means ± SD, medians (IQR) or as numbers (percentage) as applicable. Adjustment for multiple testing was performed with the Bonferroni method. A *P* value < 0.05 was considered statistically significant.

Analysis was conducted with SPSS software (Version 25.0.0, Armonk, NY: IBM Corp). R for Macintosh, Version3.2.1 (R Core Team (2020). R: A language and environment for statistical computing. R Foundation for Statistical Computing, Vienna, Austria) was used to calculate ASD.

## Results

**Fig. 1:**
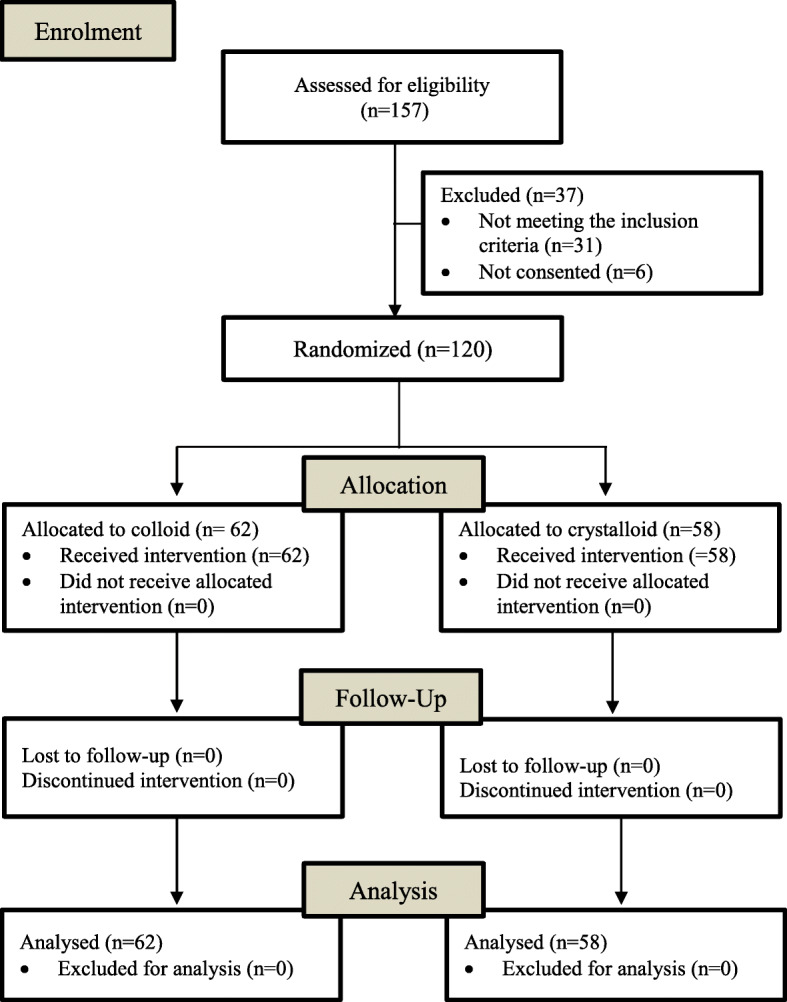
CONSORT 2010 Patient flow chart

A total of 120 patients were included between November 2012 and October 2014: 62 in the colloid group and 58 in the crystalloid group (Fig. 1). At T0 all values were measured. Overall, in the crystalloid group 96% and in the colloid group 95% of the pre-planned blood samples were collected and analyzed.

Patient’s baseline characteristics did not differ, except for height, BMI with a slightly higher BMI in the colloid group, type of surgery and CRP, also higher in the colloid group (Table 1). Duration of anesthesia and surgery were comparable between both groups. Patients assigned to crystalloid administration received a median of 3905 mL (2880–5288) crystalloids whereas patients assigned to the colloid group received 1557 mL (1207–2116) of crystalloid solution and 1250 mL (750–1938) of colloids. Blood loss, transfusion requirements and urinary output did not differ between the groups. Anesthetic management, MAP and HR did not differ between the groups. FTc, SV and CO were significantly higher in the colloid group compared to the crystalloid group (FTc: 348 ms (334–364) versus 339 ms (321–353), *p* <  0.01, SV: 91 ml (75–106) versus 76 mL (64 to 90), *p* <  0.01 and CO: 6.2 ± 1.5 L.min^− 1^ versus 5.4 ± 1.2 L.min^− 1^, *p* <  0.01). The number of patients requiring vasopressor support was comparable between groups. The incidence of postoperative ICU admissions did not differ (62% in the crystalloid versus 58% in the colloid group, *p* = 0.71) (Table 2).

**Table 1:** Baseline Characteristics

	**Crystalloids**	**Colloids**	***ASD***
	**(*n* = 58)**	**(*n* = 62)**	
**Age,** ***yrs***	57 ± 14	56 ± 14	0.14
**Weight,** ***kg***	77 ± 13	80 ± 14	0.17
**Height,** ***cm***	174 ± 10	172 ± 8	0.24
**BMI,** ***kg.m***^***− 2***^	25 ± 4	27 ± 4	0.37
**Gender,** ***No. (%)***			0.22
Men	37 (64)	33 (53)	
Women	21 (36)	29 (47)	
**ASA Score,** ***No. (%)***			0.19
I	9 (15.5)	10 (16)	
II	40 (69)	48 (77)	
III	9 (15.5)	4 (7)	
**Medical History,** ***No. (%)***			
Pulmonary Disease	4 (7)	2 (3)	0.17
Cardiovascular Disease	24 (42)	25 (40)	0.02
Diabetes Type I	0 (0)	0 (0)	< 0.001
Diabetes Type II	5 (7)	3 (5)	0.15
**Type of Surgery,** ***No. (%)***			0.22
Colorectal	25 (43)	16 (26)	
Liver	21 (36)	34 (55)	
Pancreatic	12 (21)	12 (19)	
**Preoperative Laboratory Values**			
CRP, mg.dL^−1^	0.25 ± 0.28	0.33 ± 0.33	0.24

Patient characteristics data are presented as means ± SD or as counts for the categorical outcomes

*Abbreviations*: *ASD* absolute standardized differences; absolute difference in means or proportions divided by the pooled SD; ASD values of 0.2, 0.5, and 0.8 represent small, median, and large differences

*BMI* body mass index, *m* male, *f* female, *ASA* American Society of Anesthesiologists, *CRP* C-reactive protein, *SD* standard deviation

**Table 2:** Intraoperative Data

	**Crystalloids** **(*n*** **= 58)**	**Colloids** **(*n*** **= 62)**	***p - Value***
**Duration of Anesthesia,** ***min***	330 ± 121	307 ± 117	0.29
**Duration of Surgery,** ***min***	281 ± 118	250 ± 113	0.14
***Fluid management***			
**Total Fluid Intake,** ***mL***^***a***^	4519 (3382–4824)	3247 (2495–4210)	< 0.001°
**Crystalloid,** ***mL***	3905 (2880–5288)	1557 (1207–2116)	< 0.001°
**Colloid,** ***mL***	0 (0–0)	1250 (750–1938)	< 0.001°
**Estimated Blood Loss,** ***mL***	500 (100–1000)	400 (100–875)	0.23
**Transfusion** ***yes/no (%)***	3/55 (5/95)	6/56 (10/90)	0.58
**Urinary Output,** ***mL***	350 (300–500)	405 [250, 615]	0.82
***Anesthesia Management***			
**Fentanyl,** ***mcg***	1000 [800, 1200]	850 (623–1223)	0.39
**TWA Et Sevoflurane,** ***%***	1.8 (1.6–1.9)	1.8 (1.6–2.0)	0.88
**Core Temperature,** ***°C***	36.4 ± 0.4	36.4 ± 0.5	0.98
**ICU Admission** ***yes/no (%)***	36/22 (62/38)	36/26 (58/42)	0.71
***Hemodynamic***			
**TWA MAP,** ***mmHg***	77 ± 6	75 ± 8	0.77
**TWA HR,** ***beats.min***^***−1***^	73 ± 12	70 ± 11	0.30
**TWA FTc,** ***ms***	339 (321–353)	348 (334–364)	0.009°
**TWA SV,** ***mL***	76 (64–90)	91 (75–106)	< 0.001°
**TWA CO,** ***L.min***^***− 1***^	5.4 ± 1.2	6.2 ± 1.5	0.002°
**Phenylephrine** ***yes/no (%)***	54/4 (93/7)	53/9 (85/15)	0.24

Intraoperative data are presented as means ± SD, medians (IQR) or as counts for the categorical outcomes. Means were compared with an unpaired two-sided t-tests or Mann-Whitney-U tests as appropriate, medians with Wilcoxon rank-sum tests and counts with chi-square or Fisher’s exact tests. ° represents statistical significance (*P* < 0.05)

*Abbreviations*: *Et* end tidal, *ICU* intensive care unit, *TWA* time weighted average, *MAP* mean arterial pressure, *HR* heart rate, *FTc* corrected flow time, *SV* stroke volume, *CO* cardiac output, *SD* standard deviation

^a^ Total fluid intake includes baseline, fluid boluses, antibiotics, analgesics and additional fluid, administered at the discretion of attending anesthesiologist

**Fig. 2:**
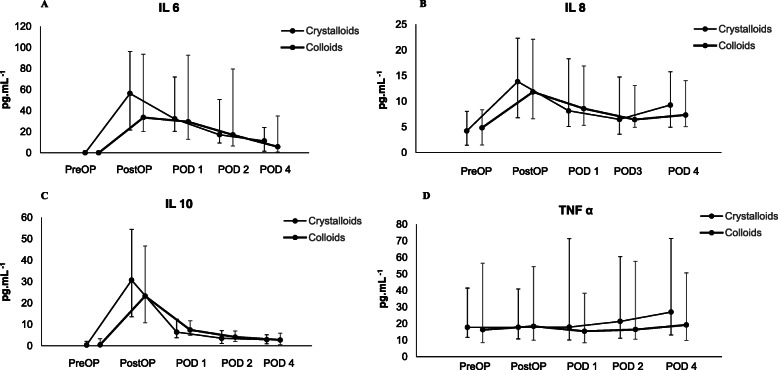
**a**-**d**: Pro- and anti-inflammatory cytokines IL 6, IL 8, IL 10 and TNF α over time. 2 A: IL 6, 2 B: IL 8, 2 C: IL 10 and 2 D: TNF α. Data are presented as medians (IQR). Abbreviations: IL – interleukin, TNF α – tumor necrosis factor alpha, POD – postoperative day

Baseline values of IL 6, IL 8, IL 10 and TNF α in the crystalloid group were comparable to values in the colloid group (IL 6: 0.00 pg.ml^− 1^ (0.00–2.04) versus 0.00 pg.mL^− 1^ (0.00–1.75), *p* = 0.95; IL 8: 4.22 pg.mL^− 1^ (1.45–8.03) versus 4.83 pg.mL^− 1^ (1.47–8.35), *p* = 0.75; IL 10: 0.31 pg.mL^− 1^ (0.00–3.30) versus 0.43 pg.mL^− 1^ (0.00–2.02), *P* = 0.71; TNF α: 17.69 pg.mL^− 1^ (11.6–41.46) versus 16.27 pg.mL^− 1^ (8.58–56.62), *p* = 0.52). Immediate postoperative values of IL 6, 8 and 10 were significantly higher compared to baseline values in both groups (*p* <  0.01 for all measurements) while TNF α did not show any significant increase in the crystalloid (*p* = 0.23) and the colloid group (*p* = 0.13) (Fig. 2). AUCs of IL 6, IL 8, IL 10 and TNF α did not differ significantly between the groups (Table 3).

**Table 3:** Areas under the curve of inflammatory markers

	**Crystalloids** **(*n* = 58)**	**Colloids** **(*n* = 62)**	***P***
**AUC IL 6,** ***pg.mL***^***− 1***^***.d***	124.3 (66.4–230.6)	104.1 (42.2–240.6)	0.60
**AUC IL** ***8, pg.mL***^***− 1***^***.d***	37.6 (25.2–84.3)	35.9 (23.5–55.1)	0.46
**AUC IL 10,** ***pg.mL***^***− 1***^***.d***	47.5 (27.1–71.9)	39.4 (25.3–66.8)	0.68
**AUC TNF α,** ***pg.mL***^***− 1***^***.d***	88.7 (51.48–208.2)	72.0 (45.5–224.8)	0.47
**AUC WBC,** ***G.L***^***−1***^***.d***	34.1 (29.8–45.7)	37.5 (31.4–43.9)	0.67
**AUC CRP,** ***mg.dL***^***− 1***^***.d***	21.71 (13.67–30.55)	22.06 (14.83–30.29)	0.83
**AUC PCT,** ***ng.mL***^***− 1***^***.d***	1.04 (0.52–1.77)	0.84 (0.62–1.39)	0.23
**AUC LBP,** ***mcg.L***^***− 1***^***.d***	73.81 (53.95–90.17)	69.09 (52.95–87.32)	0.64

Table 3: Areas under the curve of IL 6, IL 8, IL 10 and TNF α as well as WBC, CRP, PCT and LBP are presented as medians (IQR). Medians were compared with Wilcoxon rank-sum tests.

*Abbreviations*: *IL* interleukin, *TNF α* tumor necrosis factor alpha, *WBC* white blood cells, *PCT* procalcitonin, *LBP* lipipopolysaccharide-binding protein

WBC values at baseline were 4.7 G.L^− 1^ (3.7–6.0) in the crystalloid versus 5.0 G.L^− 1^ (3.9–6.0) in the colloid group (*P* = 0.50). CRP, PCT and LBP baseline values were also comparable in both groups (CRP: 0.16 mg.dL^− 1^ (0.06–0.36) versus 0.24 mg.dL^− 1^ (0.13–0.47), *P* = 0.05; PCT: 0.05 ng.mL^− 1^ (0.03–0.06) versus 0.04 ng.mL^− 1^ (0.03–0.06), *P* = 0.51; LBP: 5.32 mcg.L^− 1^ (4.26–6.61) versus 5.46 mcg.L^− 1^ (4.10–6.81), *p* = 0.91).

**Fig. 3:**
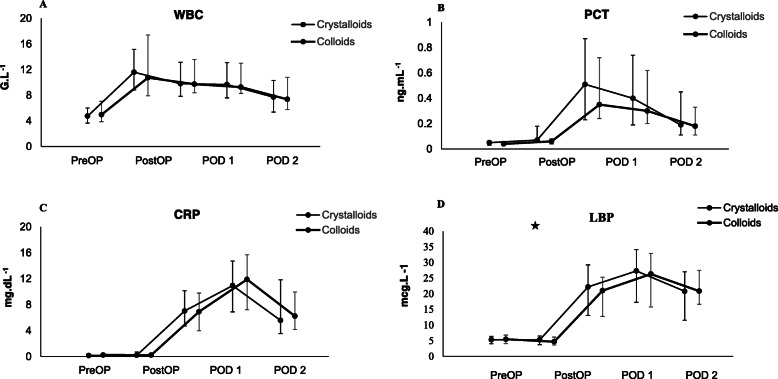
**a**-**d**: Inflammatory markers WBC, CRP, PCT and LBP over time. **a**: WBC, **b**: CRP, **c**: PCT and **d**: LBP. Data are presented as medians (IQR). Abbreviations: WBC – white blood cells, CRP – C-reactive protein, PCT – procalcitonin, LBP – lipopolysaccharide-binding protein, POD – postoperative day. ★ represents significant difference in LBP in the immediate postoperative period between the crystalloid and the colloid group (*P* = 0.38)

Immediate postoperative values of WBC and PCT were significantly higher compared to the baseline values in both groups (*P* <  0.01 for all measurements) (Fig. 3).

The AUCs for WBC, CRP, PCT and LBP for the time periods from T1 to T4 did not differ significantly between the groups (Fig. 3, Table 3). However, LBP showed significantly higher levels in the crystalloid group in the immediate postoperative period compared to the colloid group (5.3 mcg.L^− 1^ (4.0–6.7) versus 4.7 mcg.L^− 1^ (3.3–5.7), *p* = 0.04). At all other postoperative time points there were no significant differences between the groups (Fig. 3).

## Discussion

This trial is a sub-study of a large multi-center randomized trial evaluating the effect of goal-directed crystalloid versus goal-directed colloid fluid administration on a composite of serious complications after moderate to high-risk open abdominal surgery. The overall trial concluded that colloids did not decrease the composite of major complications [15]. Our results are in concordance, as they did not show any differences in perioperative pro- and anti-inflammatory markers between a crystalloid and a colloid fluid regimen.

Despite multimodal care and enhanced recovery programs it still remains challenging to blunt the inflammatory response to surgery [18]. Systemic inflammation after abdominal surgery impairs outcome and therefore many attempts have been made to alter the inflammatory response [10].

Several factors influence the perioperative inflammatory response such as the underlying disease, type and invasiveness of surgery as well as type of anesthesia [19–22]. The most important factor is the magnitude of surgical trauma and tissue damage, which induce proliferation and activation of immune competent cells, in turn triggering cytokine- and inflammatory marker release [19]. So far, very few trials have specifically investigated the influence of fluid therapy and differences in terms of the type of fluid on the extent of inflammatory marker release.

To investigate the potential influence of goal-directed 6% hydroxyethyl starch versus a lactated Ringer’s solution fluid regimen on inflammatory response, pro- (IL 6, IL 8 and TNF α) and anti-inflammatory cytokine (IL 10) serum levels were measured during the perioperative period. Additionally, we measured WBC, CRP, PCT and LBP.

Generally, the most commonly measured biomarkers are CRP and WBC [23]. If levels of CRP are above 10 mg.dL^− 1^ after postoperative day four a postoperative infection can be suspected [24]. CRP levels in our study groups increased on the first postoperative day, dropping on the fourth postoperative day to nearly 8 mg.dL^− 1^ in both study groups.

WBCs are an imprecise marker to detect postoperative complications after major abdominal surgery [10]. A more sensitive parameter in predicting postoperative complications after major abdominal surgery is IL 6 [10]. Surgical trauma and hypoperfusion of the colon are main sources of IL 6 release in colorectal surgery [25]. Noblett demonstrated that GDT during elective colorectal surgery significantly reduced IL 6 levels in comparison to a control group [7]. Yates showed no differences of IL 6 and IL 10 levels between goal-directed colloid and crystalloid fluid therapy during the first 24 h in a subgroup of patients undergoing colorectal surgery [26]. Although, patients in the crystalloid group received significant more volume amount as compared to the colloid group, there was no significant difference in hemodynamic variables [26]. Our patients showed similar courses of IL 6 and IL 10 levels in the immediate postoperative period. In contrast to the trial of Yates, who measured cytokine levels up to the first 24 h after surgery, we extended our measurement period to four postoperative days. We showed comparable circulating IL 6 and IL 10 levels between a GDT crystalloid and colloid administration. These surrogates of inflammatory response imply that gut perfusion during surgery was well preserved with both types of fluid and suggest that the type of fluid might be of minor importance as long as the fluid is administered in a goal-directed fashion.

The fact that TNF α levels in both groups remained stable over the entire measured period further supports our theory. The course of TNF α levels during the perioperative period was in accordance with the study of Szakmany, in which fluid therapy was guided with PiCCO versus central venous pressure in major abdominal surgery in patients at risk for postoperative complications [27]. As TNF α per se triggers glycocalyx degradation [9], we anticipate that TNF α did not influence glycocalyx shedding and thus possible fluid shifts in our study population.

PCT is an early predictive marker for systemic inflammation after abdominal surgery [28]. Values above 1 ng.mL^− 1^ are associated with postoperative complications such as pneumonia or anastomotic leakage [29]. In our study median PCT levels did not exceed 1 ng.mL^− 1^ at any measured time point. These results are in concordance with our main study, where infectious complications rate were held low and did not differ between the groups [15]. Moreover, as PCT production can also be induced by tissue hypoperfusion, we might assume that goal-directed fluid administration contributed to low PCT values by optimizing cardiac performance [30].

Furthermore, we measured LBP, a prognostic marker for bacterial infections [31]. Patients in the crystalloid group showed significantly higher levels immediately after surgery; however, the measured values remained within the normal range. Therefore, this difference is most likely not to of clinical importance.

The vascular endothelium is one of the earliest sites involved in the inflammatory response syndrome. An adequate perioperative fluid management has a major impact on the integrity of the glycocalyx [32]. With goal-directed fluid management individualized and time appropriate fluid resuscitation can be achieved, enabling preservation of endothelial surface layer and sufficient organ perfusion, thus improving postoperative outcomes after major surgery [33].

Patients in the colloid group received significantly less fluid (1272 mL), confirming the previously published fluid sparing effect of colloids [13]. However, clinical significance of this difference may be questionable during a perioperative period of nearly five hours. Further, our hemodynamic data showed significantly higher values of SV and CO with colloid administration, though the absolute difference of 15 mL in SV most likely has only limited clinical relevance. It might very well be that the significant differences in SV and CO are the result of our number of patients included in this substudy.

First limitation of our study is that we measured inflammatory markers that reflect the inflammatory response as surrogates and not direct markers of glycocalyx degradation like syndecan-1. Therefore, we cannot draw any conclusions about the preservation of the endothelial surface layer in our patients. Secondly, we did not control postoperative fluid management during the postoperative follow-up period. A further limitation is the time between patient enrolment and submission of our current results. Due to the fact that the main trial has been published recently a delay of our submission occurred [15]. Nevertheless, our results can still be extrapolated to current clinical practice.

## Conclusion

In summary, goal-directed hydroxyethyl starch administration did not attenuate the inflammatory response, expressed by cytokine levels of IL 6, IL 8, IL 10 and TNF α in patients undergoing moderate to high-risk open abdominal surgery. WBC, CRP and PTC values did not differ between the different fluid regimes as well.

## Data Availability

The datasets used and/or analysed during the current study are available from the corresponding author on reasonable request. barbara.kabon@meduniwien.ac.at

## Abbreviations

ASA: American Society of Anesthesiologists
ASD: Absolute Standardized Difference
AUC: Area Under the Curve
BMI: Body Mass Index
CO: Cardiac Output
CRP: C-Reactive Protein
ECG: Electrocardiography
EF: Ejection Fraction
ELISA: Enzyme-Linked Immunosorbent Assay
etCO_2_: End-tidal Carbon Dioxide
FTc: Corrected Flow Time
GDT: Goal-directed Therapy
HR: Heart Rate
IBW: Ideal Body Weight
ICU: Intensive Care Unit
IL: Interleukin
IQR: Interquartile Range
LBP: Lipopolysaccharide-Binding Protein
MAP: Mean Arterial Pressure
PACU: Post-Anesthetic Care Unit
PCT: Procalcitonin
PiCCO: Pulse Contour Cardiac Output
SPSS: Statistical Package for the Social Sciences
SV: Stroke Volume
TNF α: Tumor Necrosis Factor Alpha
TWA: Time Weighted Average
WBC: White Blood Cell Count
